# Multistage microrobots with pH-responsive release of platelet membrane–coated nanoparticles

**DOI:** 10.1126/sciadv.aee6534

**Published:** 2026-07-15

**Authors:** Rujie Sun, Junyoung Kim, Yixuan Leng, Yuyang Zuo, Jungmin Kim, Ruoxiao Xie, Tao Yang, Liyun Ma, Xin Song, Jiaxiang Ma, Jinmyoung Joo, Yoon-Kyoung Cho, Molly M. Stevens

**Affiliations:** ^1^ Department of Materials, Department of Bioengineering, and Institute of Biomedical Engineering, Imperial College London, London SW7 2AZ, UK.; ^2^ Department of Physiology, Anatomy and Genetics, Department of Engineering Science, Kavli Institute for Nanoscience Discovery, University of Oxford, Oxford OX1 3QU, UK.; ^3^ School of Electronics & Computer Science, University of Southampton, Southampton SO17 1BJ, UK.; ^4^ Department of Biological Engineering, College of Engineering, Konkuk University, Seoul 05029, South Korea.; ^5^ Department of Biomedical Engineering, Ulsan National Institute of Science and Technology, Ulsan 44919, South Korea.

## Abstract

Targeted drug delivery in the gastrointestinal tract remains challenging because therapeutics must overcome multiple hierarchical barriers before reaching diseased tissue. Here, we present a multistage delivery platform that integrates magnetic microrobots, a pH-responsive protective coating, and platelet membrane–coated nanoparticles (PNPs) in one platform. A fillable design enables the formation of an internal magnetic layer for microrobot actuation, while the pH-responsive coating protects the cargo during transit and selectively degrades upon pH change, releasing cancer cell–targeting PNPs. In an in vitro colon cancer model that reproduces key gastrointestinal features, including flow, pH variation, and villi-like structures, this strategy increased nanoparticle retention and enhanced cancer cell cytotoxicity compared to nanoparticles administered alone. Ex vivo studies in porcine stomach and intestine further demonstrated robust locomotion on compliant and folded tissue surfaces. These results establish an environment-responsive hierarchical delivery strategy for more precise oral delivery in complex gastrointestinal settings.

## Teaser

A multistage microrobot system uses bioinspired nanoformulations for improved precision intestinal drug delivery.

## INTRODUCTION

Conventional drug delivery methods, such as oral administration and intravenous injection, rely on the systemic dispersion of therapeutics that leads to poor localization in diseased areas and high risk of off-target side effects. In contrast, targeted drug delivery systems are carefully engineered to navigate biological barriers and release therapeutic agents precisely at specific sites, enhancing therapeutic efficacy and minimizing side effects (*1*). With advancements in micro- and nano-technology, several innovative delivery techniques have emerged, notably enhancing the performance of therapeutic strategies, including targeted catalytic immunotherapy (*2*), stem cell transplantation (*3*), and chemo-photothermal therapy (*4*).

Various drug delivery approaches, spanning from the nanoscale to the macroscale, have been used to address biological barriers, including cellular membranes and organ systems. In this context, nanomedicines have gained considerable attention due to their high surface area–to–volume ratio, structural tunability, and ease of functionalization. Engineered nanoparticles, in particular, stand out as promising drug carriers capable of overcoming biological barriers and achieving specific disease targeting (*5*). Their size allows them to pass through cellular barriers via mechanisms such as endocytosis, transcytosis, and passive diffusion, which are augmented by the enhanced permeability and retention effect in tumors.

At the nanoscale, targeting specificity can be improved through nanoparticle surface modification strategies such as chemical coating, bioconjugation with carbohydrates, antibodies, and peptides, and/or biomimetic coating with natural cell membranes (*6*–*9*). In particular, coating with natural cell membranes, derived from platelets, erythrocytes, macrophages, cancer cells, and bacteria, can confer the biological properties of the source cell membrane to the nanoparticle surface, including homotypic targeting, immune evasion, and prolonged circulation life (*10*). This approach has been successfully used in therapies for cancer (*11*), thrombosis (*12*), heart disease (*13*), detoxification (*14*), and vaccination (*15*). Specifically, platelets are known for their high binding affinity to cancer cells through specific membrane receptor-ligand interactions, such as integrin α6β1 on platelets with ADAM-9 on cancer cells and P-selectin on platelets with PSGL-1 or CD24 on cancer cells (*16*). This observation has led to the active development of platelet membrane–coated nanoparticles (PNPs) to target cancer cells and the tumor microenvironment in therapeutic applications (*17*). However, current systemic and tumor-targeted administration methods yield limited targeting efficacy, with less than 0.7% of the total administered dose accumulating at the intended disease site (*18*). This inefficiency is primarily due to the complex environment within the human body, characterized by rapid hemodynamics, immune clearance, the reticuloendothelial system, renal filtration, and multiple structural barriers, including the epithelium, the endothelium, and the extracellular matrix (*19*).

Further targeting capabilities can be achieved with the integration of microscale devices. For example, advanced microelectronics and various propulsion mechanisms enable multifunctional capabilities such as real-time sensing, precise navigational control, and on-demand drug release (*20*). As a representative example of these systems, microrobots have shown great potential for site-specific drug delivery. Unlike nanoparticles, which passively rely on biochemical interactions for targeting, microrobots can be actively guided within the body using external fields, such as magnetic (*21*), optical (*22*), and acoustic stimuli (*23*); or propelled by self-actuated mechanisms like catalytic reactions (*24*) and microorganism-based motion (*25*). Magnetic control is especially attractive due to its minimally invasive deep tissue penetration, enabling programmable navigation and accumulation at specific sites with high precision (*21*). Moreover, microrobots provide great design flexibility for integrating advanced loading strategies, including microfluidic filling, making them suitable for encapsulation of various pharmaceutical modalities (*26*).

Despite these advancements in micro- and nanoscale drug delivery systems, single-scale delivery approaches often struggle to achieve sufficient therapeutic payload localization because oral delivery must overcome hierarchical physiological barriers spanning macro- to micro-/nanoscales (*27*–*29*). Along the gastrointestinal (GI) tract, pharmaceutical modalities encounter dynamically changing physiological microenvironments, including varying pH levels, enzymatic activity, thick mucus layers, and peristaltic motion, posing substantial obstacles for oral drug administration. Orally administered drugs must resist gastric acidity, penetrate the intestinal mucus layer, and successfully accumulate at tumor sites within the intestines, all while avoiding enzymatic degradation and dilution (*30*). Microrobots are highly effective in navigating through macroscale barriers; however, they are limited in penetrating nanoscale biological interfaces. In contrast, nanoparticles can traverse nanoscale interfaces, such as cellular membranes, but lack adequate macroscale targeting capabilities. These complementary strengths motivate a versatile multistage drug delivery approach that integrates macro-/microscale navigation with nanoscale interfacing and microenvironment-responsive release within a hierarchical platform. Such a framework is broadly applicable across GI diseases and conditions and is particularly relevant in contexts where site-localized intestinal delivery is desired, such as colon cancer.

Herein, we developed a multistage drug delivery platform that integrates magnetic microrobots, pH-responsive sealing, and PNPs into a single functional system. Our system, termed p-bots (PNP-loaded microrobots), combines the macroscale navigational capabilities of magnetic microrobots with the platelet-mimetic targeting properties of PNPs. The fillable microrobot architecture enables a fillable chamber–based magnetization strategy, allowing the formation of an internal magnetic layer for actuation. In addition, the pH-responsive sealing layer protects the payload during gastric transit under acidic conditions and then degrades under intestinal-like conditions, enabling autonomous site-specific release without an external stimulus. The fillable architecture also provides flexibility to load different therapeutic cargos and pharmaceutical modalities, ranging from aqueous solutions for relatively rapid release to hydrogel-encapsulated formulations for more prolonged release, thereby expanding the system’s therapeutic applicability. We evaluated this multistage strategy in an in vitro colon cancer model featuring a microfluidic channel with villi-like structures, pH and flow conditions that mimic the GI tract, and further validated microrobot locomotion on compliant and folded ex vivo porcine GI tissues. Together, this hierarchical approach addresses the limitations of conventional single-stage drug delivery systems and expands possibilities for oral delivery strategies to target complex and difficult-to-access disease sites.

## RESULTS

### Design and fabrication of pH-responsive and magnetic microrobots for multistage targeted drug delivery

Oral administration is a noninvasive, easy-to-use, and patient-compliant route with strong potential for colon cancer treatment, as it enables localized treatment directly within the GI tract (*31*). However, achieving site-specific drug accumulation via oral delivery remains challenging due to the anatomical complexity of the GI tract and its dynamic environment. To address these challenges, we developed a multistage system featuring a unique combination of cancer cell–targeting PNPs encapsulated in a pH-responsive microrobotic carrier.

**Fig. 1.**
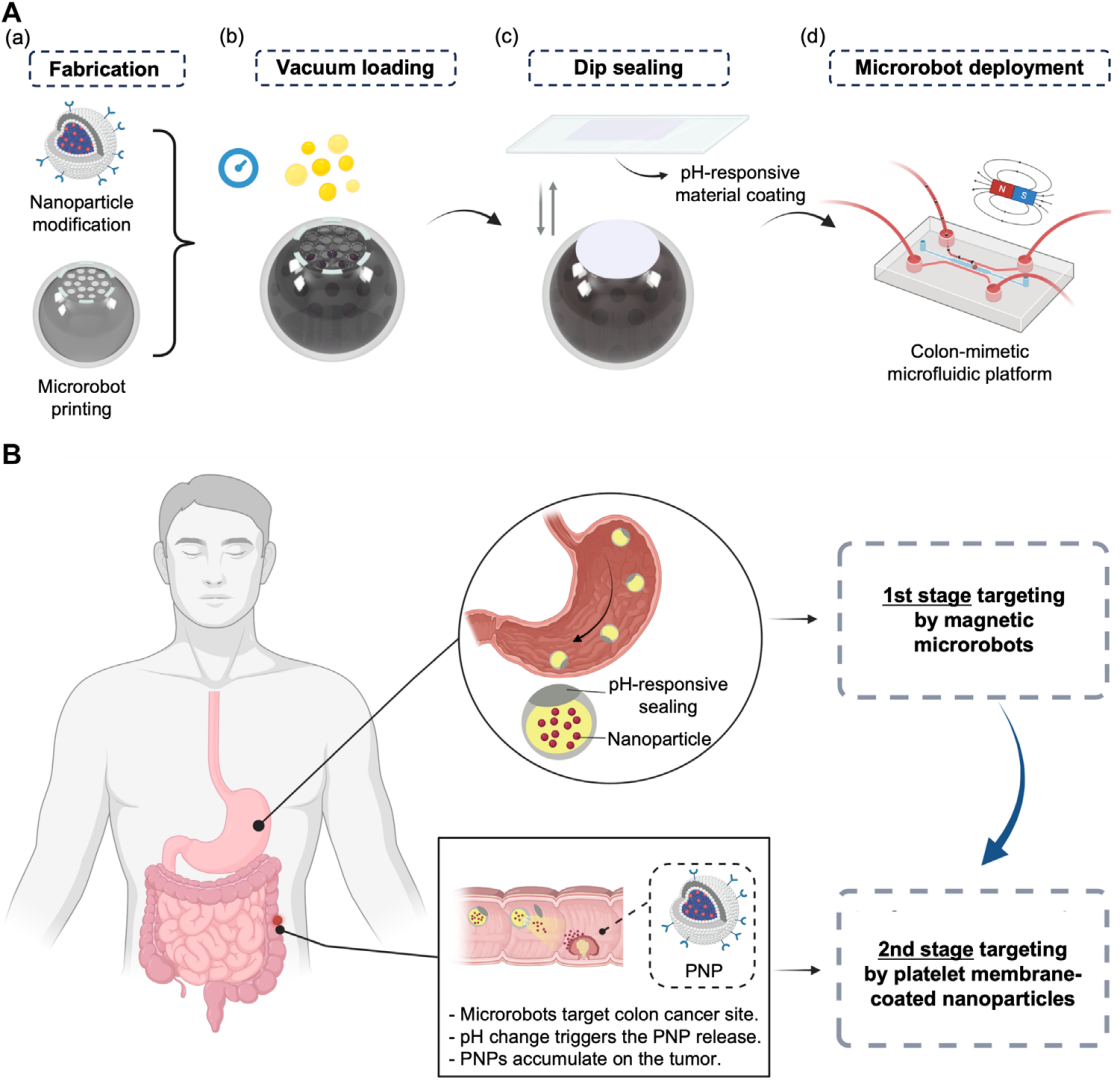
Schematic illustration of the proposed multistage targeted drug delivery system. (**A**) The fabrication and deployment workflow consists of four main steps: (a) synthesizing the modified nanoparticles and three-dimensional (3D) printing the microrobots; (b) loading the platelet membrane–coated nanoparticle (PNP)–based cargo into the microrobot chamber; (c) sealing the loaded microrobot with a pH-responsive polymer; and (d) deploying the loaded and sealed microrobot into the simulated GI environment, where it is navigated and controlled by an external magnetic field. (**B**) The envisioned application for colon cancer treatment would feature a two-stage targeted delivery approach using microrobots and PNPs. The first stage of targeting is achieved through magnetically controlled motion that enables precise navigation within the GI tract. The second stage of targeting is accomplished by platelet membrane–coated nanoparticles (PNP), which specifically bind to cancer cells. Figures were created in BioRender. Cho, Y. (2026) https://BioRender.com/7dqtc5s and https://BioRender.com/m4royby.

Figure 1A summarizes the fabrication process of magnetic microrobots for multistage targeted drug delivery. First, fillable microrobots are printed using two-photon polymerization (2PP), and biomimetic nanoparticles are prepared. The nanomedicines are introduced into the microrobot chamber via vacuum loading. Once fully loaded and cleaned, the microrobots are immediately sealed with a pH-responsive material, composed of a copolymer of methacrylic acid and ethyl acrylate, using dip coating to minimize solution evaporation. We validated the platform using an in vitro colon-mimetic microfluidic model. Precise targeting is achieved by capitalizing on the micro- and nanoscale features of the system. In stage 1, microrobot navigation is controlled by an externally applied three-dimensional (3D) magnetic field, enabling precise movement through a microfluidic channel that mimics the GI tract. The microrobot can be navigated toward a target site housing a colon cancer spheroid. The local pH change at the target site triggers the degradation of the sealing layer, releasing the nanoparticle solution. The second stage of targeting is achieved through the action of PNPs, which exhibit a high affinity toward colon cancer cells, thereby enhancing therapeutic delivery.

As illustrated in Fig. 1B, we envision applying this magnetic microrobot system for the oral administration of colon cancer nanomedicines. Its pH-responsive sealing would effectively protect the drug from the acidic environment during transit through the GI tract. Unlike previously reported systems that rely on thermal or light-triggered release mechanisms (*32*, *33*), which require external control units and face issues such as poor tissue penetration, our system harnesses the natural pH gradient of the GI tract. The microrobots would remain stable and selectively release the payload upon encountering the intestine environment.

### Design and characterization of microrobots for stage 1 targeting

**Fig. 2.**
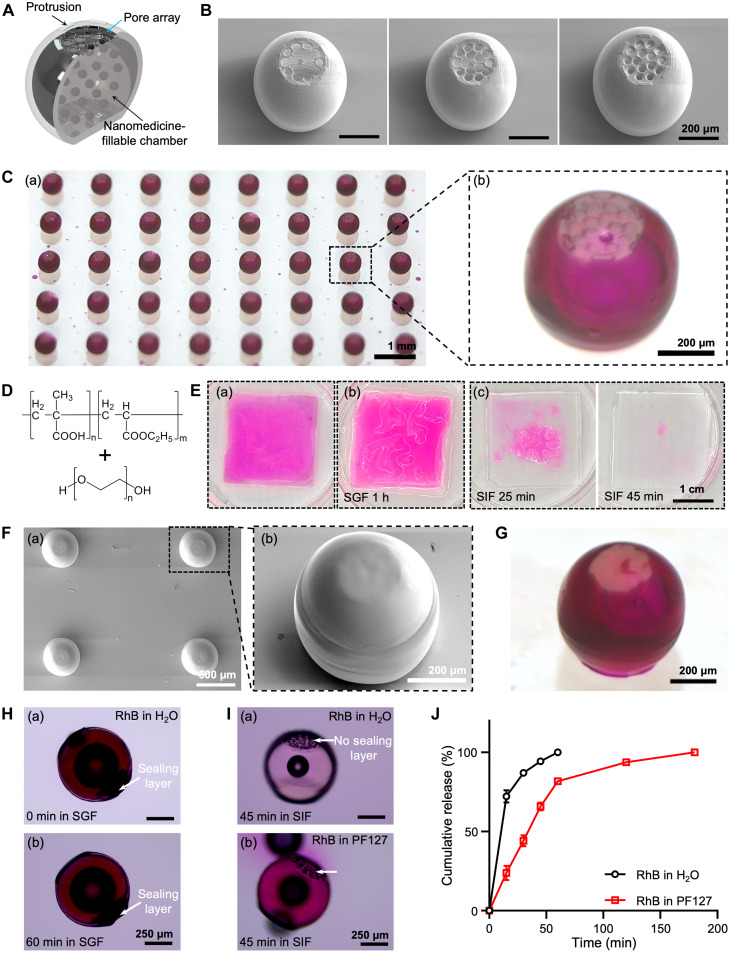
Characterization of the pH-responsive magnetic microrobot. (**A**) Schematic representation of the fillable microrobot configuration, featuring a top lid with protrusions and a pore array, along with an internal chamber designed for cargo loading. (**B**) Optimization of the 3D printing process with varying slice thicknesses of 3.5, 2.5, and 1.5 μm (from left to right). (**C**) Rhodamine B aqueous solution (RhB-H_2_O) is loaded into the microrobot chamber using a vacuum loading method. All microrobots within the array are successfully loaded without compromising the protrusion features on the top lid. (**D**) A pH-responsive ink, based on a copolymer derived from methacrylic acid, ethyl acrylate, and PEG, is used to seal the microrobot. (**E**) pH-responsive degradation of the copolymer coating on glass slides, visualized with RhB. (a) Uniform copolymer coating on the glass slide before immersion. (b) The film remains intact after 1 hour in simulated gastric fluid (SGF). (c) After transfer to simulated intestinal fluid (SIF), the film begins to dissolve after 25 min and is nearly completely dissolved after 45 min. (**F** and **G**) Validation of the sealing performance of the microrobot using (F) scanning electron microscope and (G) optical microscopy images. (**H**) Stability of sealed microrobots loaded with Rhodamine-B solution in SGF at 37°C. The sealing layer remains intact after 60 min. (**I**) Comparison of the release process for microrobots loaded with (a) RhB-H_2_O and (b) RhB suspended in Pluronic F-127 (PF127) solution (RhB-PF127) in SIF at 45 min. (**J**) Release profiles based on absorbance measurements from RhB-H_2_O and RhB-PF127 demonstrate that the addition of PF127 markedly delays the release process, offering controlled release over time (*n* ≥ 3 independent samples). Data represent mean ± SD.

The first targeting stage combines magnetic localization of the microrobot with pH-triggered release of the nanoparticle payload. We designed the fillable microrobot with a spherical shape to enable multimodal motion, including rolling, spinning, and wobbling (*32*). The diameter of 550 μm was selected as a practical compromise among internal loading capacity, fabrication fidelity, sealing reliability, and fabrication efficiency. This size provides sufficient internal space for cargo loading and formation of the internal magnetic layer, while remaining compatible with high-resolution fabrication of the 40-μm pore array used for vacuum loading and the 5-μm protrusion array for precise sealing (Fig. 2A). These microrobots were fabricated using high-resolution 3D printing based on 2PP with biocompatible resin IP-Q, which provides the submicrometer precision necessary for printing complex microscale features. IP-Q has been reported to be cytocompatible after standard postprocessing and has been used in 2PP-printed microdevices and microstructured platforms for cell-based studies, including long-term coculture with 3D cell spheroids (*34*, *35*). In 3D printing, the structure is sliced into layers and printed sequentially; optimizing printing efficiency requires balancing slice thickness and resolution. Thicker slices reduce the total number of layers and printing time, but can compromise structural resolution. We evaluated the effect of slice thicknesses of 3.5, 2.5, and 1.5 μm on printing outcomes via scanning electron microscope (SEM) images, which revealed that only the 1.5-μm slice thickness successfully reproduced all the microrobot features, including the top lid, while thicker settings failed to print the top lid (Fig. 2B).

We successfully printed an array of microrobots and established an optimized cleaning and postcuring process, resulting in a clean internal chamber as confirmed by clear microstructural features (fig. S1). After printing and postprocessing, the microrobots undergo a fillable chamber–enabled magnetization procedure. Briefly, a suspension of Neodymium Iron Boron (NdFeB) microparticles is loaded into the internal chamber by centrifugation, allowing particles to sediment and form a stable magnetic layer at the chamber bottom after solvent drying. This magnetic layer is subsequently isolated from the drug payload by a thin polydimethylsiloxane (PDMS) barrier layer, and the microrobots are then postmagnetized using an impulse magnetizer to define a consistent magnetic orientation across the array. Following magnetization, cargo is vacuum filled through the lid pores, enabling uniform loading across the printed array [Fig. 2C (a)]. A Rhodamine B (RhB) aqueous solution is used as a small-molecule model drug due to its strong absorbance. The pressure from the applied vacuum efficiently drove the solution into the internal chambers, and subsequent spinning removed any excess solution, yielding clean particle surfaces with the remaining lid structure clearly distinguishable [Fig. 2C (b)]. Notably, the loading process at 4°C efficiently prevents fast evaporation of the small internal volume (∼60 nl), preserving drug content for further experiments.

Following drug loading, all microrobots were successfully sealed using a pH-responsive polymer layer. This measure would ensure cargo retention under gastric conditions and controlled release in the intestines. As illustrated in Fig. 2D, the sealing material, a copolymer of methacrylic acid and ethyl acrylate commonly used in enteric coating for oral solid dosage forms, was further modified with 15 wt % polyethylene glycol, molecular weight 400 to enhance viscosity for uniform dip sealing. To validate the seal’s pH responsiveness, copolymer-coated glass slides were immersed in simulated gastric fluid (SGF), which showed no visible dissolution after 1 hour [Fig. 2E (a) and (b)]. However, when transferred to simulated intestinal fluid (SIF), the copolymer coating started to dissolve after 25 min and was nearly completely dissolved by 45 min, confirming effective pH-triggered degradation under intestinal-like conditions [Fig. 2E (c)].

Dip sealing the microrobot array using the optimized copolymer layer on a glass slide, followed by air drying, resulted in a thin and continuous sealing layer in close contact with the top lids of microrobots. As shown in Fig. 2 (F and G), both SEM and optical microscopy confirmed that the pores and protrusions on the lid were fully covered with the pH-responsive polymer, ensuring complete protection of the payload. To assess the sealing stability, critical to preserve encapsulated drug doses during transit through the stomach, the loaded and sealed microrobots were immersed in SGF at 37°C. After 60 min, the sealing layer remained intact, and no leakage of RhB aqueous solution (RhB-H_2_O) was observed (Fig. 2H). In contrast, immersion in SIF triggered a gradual release of the RhB-H_2_O from the chamber with a marked color change of microrobots from dark red to light red within 45 min, indicating that most of the cargo was released [Fig. 2I (a)]. To further evaluate the robustness of the pH-responsive sealing layer under gastrointestinally relevant conditions, we expanded the release study to include intermediate pH values (pH 3, 4, 5, and 6), in addition to SGF and SIF. RhB-H_2_O–loaded, dip-sealed microrobots were incubated in each medium for 1 hour, and cargo release was quantified by measuring the absorbance of the surrounding solution. The sealed microrobots remained stable, with minimal premature leakage, in SGF and across pH 3 to 6. By contrast, substantially higher release was observed in SIF after 1 hour (fig. S2). Together, these results demonstrate that the sealing layer provides effective protection under gastric-like and intermediate pH conditions, while enabling triggered release under intestinal-like conditions.

Next, we examined a Pluronic F-127 (PF127) solution as an alternative cargo matrix to modulate the release kinetics, due to the phase change that PF127 undergoes in response to temperature. As shown in Fig. 2I (b), microrobots loaded with 1.0 wt % RhB suspended in PF127 solution (RhB-PF127) at 4°C and sealed using the same process showed a much-delayed release profile compared to RhB-H_2_O–loaded microrobots. Visual and quantitative analyses revealed that more than 80% of the cargo was released within 15 min for the RhB-H_2_O–loaded microrobot group, while the RhB-PF127–loaded group only released around 20% of cargo in the same time frame, requiring 60 min to exceed 80% cargo release (Fig. 2J). These results confirm that our microrobot system enables pH-triggered, environment-specific cargo release with tunable kinetics, suggesting strong potential for oral delivery throughout the GI tract.

### Design and characterization of platelet membrane–coated nanomedicines for stage 2 targeting

**Fig. 3.**
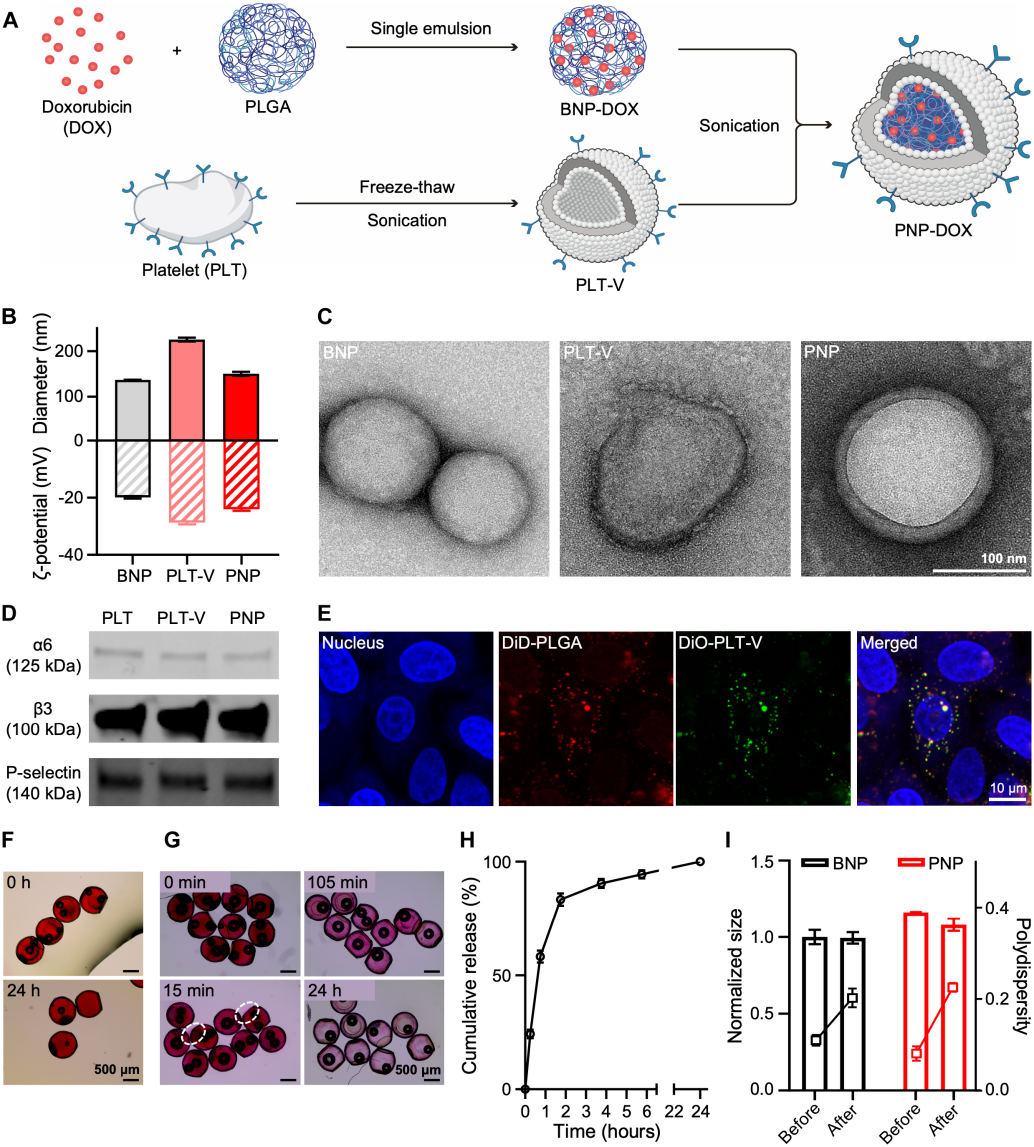
Preparation and characterization of platelet membrane–coated nanoparticles (PNP) containing doxorubicin (DOX) and characterization of PNPs loaded into and released from microrobots. (**A**) Schematic illustration of the preparation process for platelet membrane–coated nanoparticles containing DOX (PNP-DOX). (**B**) Average hydrodynamic diameter and zeta potential measurements of bare nanoparticles (BNPs), PLT membrane derivatives (PLT-Vs), and PNPs (*n* = 3 technical replicates). (**C**) Representative transmission electron microscopy images of BNPs, PLT-Vs, and PNPs. (**D**) Western blot analysis of platelet-related and tumor interaction–related surface protein expression in platelets (denoted by PLT, PLT-Vs, and PNPs). Figure assembled from three separate gels. (**E**) Fluorescence image showing internalized DiD-labeled PLGA nanoparticle core (DiD-PLGA, red) coated with DiO-stained PLT-V (DiO-PLT-V, green) in HCT116 cells (Hoechst 33342-stained nuclei, blue) (*n* = 3 independent samples). (**F**) Microrobots loaded with PNP solution showing intact sealing after 24 hours of immersion in SGF at 37°C. (**G**) Images showing gradual release from microrobots loaded with PNP solution in SIF at 37°C. The dotted circle indicates the PNP release trace (pink) forming around the lid of the microrobots. (**H**) Release profiles of PNPs from microrobots based on absorbance measurement, showing that more than 80% of the loaded PNPs in cargo were released within 105 min (*n* = 3 independent samples). Data represent mean ± SD. (**I**) Change of average hydrodynamic diameter (bar graph) and polydispersity index (PDI, line graph) for BNPs and PNPs before loading and after loading into microrobots (*n* = 3 independent samples). Data represent mean ± SD. (A) was created in BioRender. Cho, Y. (2026) https://BioRender.com/fbzhbg9.

The interaction between platelets and tumor cells in the colon cancer microenvironment is a crucial factor in tumor initiation, progression, and metastasis (*36*). On the basis of this biological phenomenon, we prepared PNPs that exhibit high targeting ability against colon cancer. To prepare doxorubicin (DOX)-loaded platelet membrane–coated biomimetic nanoparticles (PNP-DOXs), platelet membrane derivatives (PLT-Vs) were first prepared by isolating platelets from whole blood through sequential centrifugation, followed by freeze-thaw cycles and sonication. These PLT-Vs were then directly coated onto DOX-loaded bare poly(lactic-co-glycolic) acid (PLGA) nanoparticle cores (BNP-DOXs) via sonication, thereby imparting the platelet membrane properties onto the nanoparticles (Fig. 3A).

Various nanoparticle characterization methods were used to assess the extent of platelet membrane coating on the nanoparticles. According to dynamic light scattering (DLS) results, the average diameter of the bare nanoparticles (BNPs) was approximately 137 ± 0.4 nm, which increased to 150 ± 4.6 nm after coating with PLT-V, indicating an increase of approximately 13.5 nm, corresponding to the thickness of the thin PLT-V layer. The zeta potential also changed from −20 ± 0.42 mV to −24 ± 0.55 mV, which is similar to the zeta potential of PLT-Vs measured at −29 ± 0.61 mV (Fig. 3B). Transmission electron microscopy (TEM) images clearly showed that a unilamellar cell membrane enveloped the BNP cores (Fig. 3C). In addition, we used Western blot analysis to investigate the transfer of platelet-derived surface proteins onto the PNPs. Specifically, platelet-related surface proteins (P-selectin) and those involved in tumor interaction (α6, β3, and P-selectin) were identified in all platelets, PLT-Vs, and the final membrane-coated nanoparticles (Fig. 3D). Confocal fluorescence visualization verified the integrity of the platelet membrane coating around the BNPs after cellular uptake. We observed colocalization of BNPs and PLT-Vs after cellular uptake, indicating a stable core-shell structure (Fig. 3E). On the basis of previous studies, DOX was selected as a model drug to evaluate our targeted delivery system in an in vitro colon cancer model (*37*). After desalting DOX–hydrochloric acid to obtain its hydrophobic state (*38*), BNP-DOXs were prepared using a single emulsion method, achieving a loading capacity of approximately 33 ± 0.51%. After PLT-V coating, PNP-DOXs exhibited a loading capacity of 32 ± 2.0%. The release profile showed similar burst biphasic patterns for both BNP and PNP, with 64% and 66%, respectively, of the encapsulated drug being released within 24 hours in phosphate-buffered saline (PBS) at 37°C (fig. S3). Long-term storage stability was confirmed in deionized water and culture media at 4°C for 7 days. The PNPs maintained their size in both conditions, indicating high colloidal stability (fig. S4).

We then characterized the PNP release from the microrobots. The optimized PNP solution was loaded into the microrobots using the vacuum loading and dip sealing processes described previously. For the PNP-loaded microrobots (p-bots), the diameter was scaled up to 910 μm, with a chamber volume of 280 nl. This size adjustment provided a suitable volume loading, minimized solution evaporation, and reduced the risk of PNP aggregation. As shown in Fig. 3F, the p-bots immersed in SGF for 2 hours showed no noticeable changes, demonstrating both the stability of encapsulated PNP solutions and the robustness of the loading and sealing strategy across different cargo types. Subsequently, we investigated the release profile in SIF. As shown in Fig. 3G, a pink release trace formed around the p-bot lid within the first 15 min, indicating the initiation of release. Over time, the color intensity inside the chamber gradually faded, consistent with a progressive, time-dependent release profile. The release behavior was quantitatively monitored by measuring the absorbance of the released RhB-labeled PNP solution. As illustrated in Fig. 3H, approximately 25% of the encapsulated PNPs were released within 15 min, comparable to the 20% release observed from the microrobots loaded with RhB-PF127. Release then continued over time, reaching more than 80% by 105 min, indicating a time-dependent, controlled release profile from the microrobot system.

Last, we analyzed the nanoparticle size and polydispersity index (PDI) before and after loading into microrobots. The results showed that neither the BNPs nor the PNPs increased in size after loading into the microrobots, suggesting that no aggregation occurred inside the microrobots. Although the PDI of BNPs and PNPs exhibited a slight increase from 0.11 to 0.21 and from 0.10 to 0.22, respectively, the values in both cases still reflected a monodisperse distribution (Fig. 3I).

### In vitro validation in a colon cancer microfluidic model and ex vivo evaluation of microrobot locomotion

**Fig. 4.**
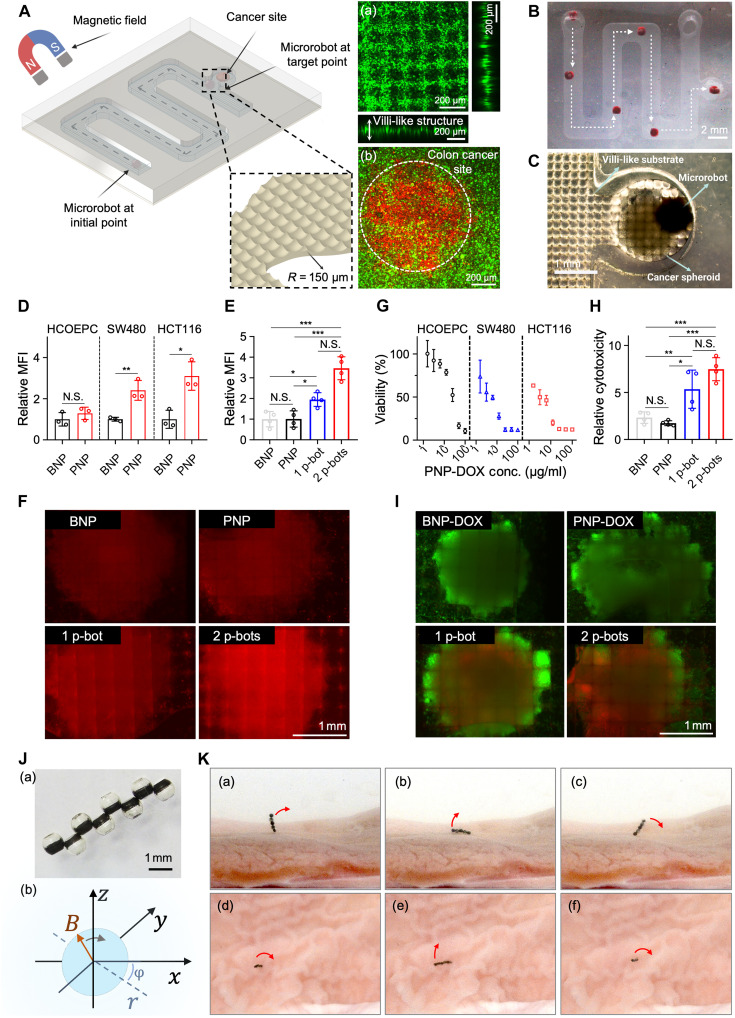
Targeting, anticancer efficacy, and ex vivo locomotion of p-bots in colon-mimetic and gastrointestinal models. (**A**) Schematic of the colon-mimetic microfluidic platform and z-stack fluorescence images of the villi-like structure consisting of normal colon epithelium cells [green, (a)] and a colon cancer spheroid placed at the target site [red area outlined white dotted circle, (b)]. (**B**) Representative image of p-bot navigation through the microfluidic channel under magnetic control. (**C**) Representative image of p-bot positioning near a colon cancer spheroid. (**D**) Flow cytometry analysis of nanoparticle adhesion to HCOEPC, SW480, and HCT116 cells, shown as relative mean fluorescence intensity normalized to the BNP group (*n* = 3 independent samples). (**E**) Relative fluorescence intensity of nanoparticles on the spheroid after 24 hours of various drug administrations, normalized to BNP conditions (*n* = 4 independent chips). (**F**) Fluorescent images of nanoparticles (red) adhered to a colon cancer spheroid. (**G**) Cell Counting Kit-8 analysis of HCOEPC, SW480, and HCT116 cells under different concentrations of PNP-DOX after 30-min targeting, washout, and 24-hour incubation (*n* = 3 independent samples). (**H**) Relative cytotoxicity comparison of colon cancer spheroids after 24 hours of various drug administrations, normalized to the nontreated conditions (*n* = 4 independent chips). BNP, PNP, and two p-bots were administered at a dose of 0.84 μg, whereas the one p-bot condition received 0.42 μg. (**I**) Fluorescent images of viability staining on the spheroid (green: Calcein AM, red: ethidium homodimer–1). (**J**) (a) The chain structure assembled from magnetized microrobots. (b) Schematic of the rotating magnetic field used to drive walking locomotion. (**K**) Time-lapse images of ex vivo locomotion on porcine tissues: side view on stomach tissue (a to c) and top view on intestine tissue (d to f). Data represent mean ± SD. Statistics are described in Materials and Methods.

We evaluated the system’s ability to target and kill colon cancer cells in two-dimensional (2D) cellular assays and in an advanced colon-mimetic microfluidic platform. First, to fabricate the microfluidic platform, we assembled a PDMS substrate with an intestinal villi-like surface structure featuring ridges with a height of 150 μm along a microchannel containing a winding path and a target site (Fig. 4A). Normal colon epithelial cells (HCOEPCs) were cultured on the substrate and then a HCT116 colon cancer spheroid was positioned at the target site. Z-stack 3D fluorescence imaging showed that HCOEPCs were evenly cultured on the substrate surface structures [Fig. 4A (a)]. The HCT116 spheroid was placed on the HCOEPC layer at the target site [Fig. 4A (b)]. We confirmed that the p-bots could navigate through the winding path (Fig. 4B and movie S1) under external magnetic field control (MFG-100-i, Magnebotix) and reach the colon cancer spheroid (Fig. 4C). To simulate drug washout by luminal flow, nanoparticles or p-bots were introduced under hydrostatic pressure–based flow for 30 min, followed by washing steps to remove unbound nanoparticles or p-bots, with an additional washing step performed 12 hours later.

We compared the targeting ability of BNPs and PNPs to various colon cells in 2D cellular assays. PNPs and BNPs exhibited comparable binding affinity for normal colonic epithelial cells. Conversely, compared to BNPs, PNPs showed 3.1 and 2.4 times higher binding to colon cancer cell lines SW480 and HCT116, respectively, confirming the colon cancer–targeting ability of the platelet membranes (Fig. 4D). In addition, blocking P-selectin on PNPs with an anti–P-selectin antibody reduced binding to HCT116 cells by ∼63.3%, while red blood cell membrane–coated nanoparticles showed binding levels comparable to BNPs (fig. S5). These results confirm that platelet membrane surface proteins, particularly P-selectin, contribute to platelet membrane–mediated tumor targeting.

We then evaluated the nanomedicine targeting ability in a colon-mimetic microfluidic platform using various configurations: BNPs, PNPs, one microrobot loaded with PNPs, and two microrobots loaded with PNPs. When 0.84 μg of nanoparticles was administered into the microfluidic platform, the amount was equivalent to the total nanoparticle load released from two microrobots (microrobot reservoir volume = 280 nl; nanoparticle stock concentration = 1.5 mg/ml). The difference in mean fluorescence intensity at the spheroid site between BNPs and PNPs was not statistically significant. However, the administration of PNPs via two microrobots resulted in a 3.4-fold higher signal compared to the administration of PNPs alone, and even with one microrobot, a 1.9 times higher signal was observed (Fig. 4, E and F). Consistent with these observations, dose-normalized quantification of the retained nanoparticle payload further confirmed the enhanced delivery efficiency of the microrobot system. Despite receiving the same administered dose (0.84 μg), the 2 p-bot group achieved ∼4.57-fold higher nanoparticle retention within spheroids compared to PNPs administered alone, corresponding to an increase in retention efficiency from ∼8.75% for nanoparticle-based delivery to ∼37.41% for robot-based delivery (fig. S6). These results indicate that using p-bots to deliver PNPs substantially improves the retention efficiency in colon cancer spheroids by mitigating washout in environments with complex structures and under flow conditions.

We then evaluated the cytotoxic effects of different nanoparticle administration methods against colon cancer cell lines following a 24-hour treatment. Under static incubation conditions, Cell Counting Kit-8 (CCK-8) analysis showed that PNP-DOXs exhibited half-maximal inhibitory concentration (IC_50_) values of 4.9 ± 0.52, 1.4 ± 0.24, and 2.0 ± 0.34 μg/ml for HCOEPC, SW480, and HCT116 cells, respectively. Compared with BNP-DOXs, the IC_50_ values for PNP-DOXs were modestly lower in the colon cancer cell lines SW480 and HCT116 (fig. S7). However, static incubation in which cells are continuously exposed to administered nanoparticles throughout the treatment period does not capture cancer selectivity and washout effects present in physiological environments. To better mimic these conditions, we performed an additional assay incorporating a washout step after a 30-min targeting phase. Under these conditions, the IC_50_ values of PNP-DOXs for all types of cells were consistently higher across all tested cell types compared with those obtained in static incubation. IC_50_ values of PNP-DOXs for colon cancer cells (SW480: 7.22 ± 5.01 μg/ml, HCT116: 8.64 ± 2.87 μg/ml) were significantly lower than those for normal epithelial cells (HCOEPC: 24.5 ± 1.35 μg/ml), indicating enhanced cancer selectivity mediated by platelet membrane–based targeting (Fig. 4G and fig. S8). These observations suggest that the platelet membrane coating promotes stable adhesion to cancer cells via specific receptor-mediated interactions (*17*), thereby enhancing selective cytotoxicity under physiologically relevant conditions.

Following the same drug administration conditions, the cytotoxicity within the colon-mimetic microfluidic platform, comprising normal colon epithelium and cancer spheroids arranged in complex structures that hinder nanoparticle delivery, was assessed using combined staining with Calcein AM and ethidium homodimer–1. Figure 4 (H and I) illustrates that PNP-DOX delivery using one or two microrobots, compared to PNP-DOXs alone, enhances cytotoxicity against colon cancer cells by 3.1- and 4.3-fold, respectively. Notably, BNP-DOXs and PNP-DOXs administered without microrobots exhibited 2.0- and 1.5-fold higher cytotoxicity toward normal colon epithelium, respectively, compared to PNP-DOXs delivered via two microrobots. However, these differences were not statistically significant (fig. S9). These results are consistent with expectations, because the extent of exposure to normal colon epithelium when BNP-DOXs and PNP-DOXs are administered alone is higher than when PNPs are released via microrobots. Our colon-mimetic platform has several limitations, including the lack of peristalsis and a mucosal layer. However, these results demonstrate an encouraging proof of concept for multistage systems that combine direct nanoparticle delivery via microrobots and efficient targeting by PNPs to reduce off-target side effects and enhance specific cytotoxicity.

To extend this proof of concept beyond the microfluidic platform and further examine the practicality of the proposed design under more physiologically relevant and larger-scale conditions, we next examined microrobot chain assembly and ex vivo locomotion on GI tissues. With a consistent magnetization orientation, each microrobot behaves as a miniature permanent magnet with defined magnetic polarity, enabling straightforward chain assembly via magnetic dipole-dipole interactions [Fig. 4J (a)]. This chain configuration supports surface “walking” locomotion under magnetic control. Specifically, the applied rotating magnetic field B [Fig. 4J (b)] rotates about a prescribed axis r lying in the xy plane. The in-plane angle φ determines the direction of microrobot translation. We further demonstrated robust locomotion in ex vivo porcine intestine and stomach tissues (movies S2 and S3), providing evidence that the proposed microrobot design remains functional in more physiologically relevant GI environments. The assembled chain maintained stable walking on compliant, uneven, and folded tissue surfaces, rather than only on idealized planar substrates. In particular, the microrobot chain was able to traverse folded stomach surfaces while also sustaining controllable locomotion on intestinal tissue (Fig. 4K), highlighting the adaptability of the design to complex GI topography. To further assess the scale discrepancy between the benchtop model and practical in vivo deployment, we performed magnetostatic simulations of a permanent-magnet configuration. The corresponding field-distance profile (fig. S10) shows that the magnetic flux density remains at the 10-mT level over 400 mm, which is above the experimentally validated actuation benchmark used in this study (5 mT at 1 Hz). These results support the feasibility of generating sufficient magnetic fields for microrobot actuation at macroscale stand-off distances relevant to practical deployment.

## DISCUSSION

The key innovation of our system lies in the integration of targeting abilities across scales. The configuration of our multiscale system addresses a critical gap in hierarchical drug delivery—here, the microrobots provide organ-scale navigation while the PNPs confer cellular targeting ability. Furthermore, the pH-triggered release mechanism responded to environmental changes such as those present in the GI tract. Our approach eliminates the need for external thermal or optical triggers, which are constrained by limited penetration in vivo. This work also introduces a fillable chamber–enabled magnetization strategy as a further methodological advance. This strategy provides a simple route to magnetic actuation while preserving visibility of cargo loading and release.

The microrobots maintained structural integrity under acidic conditions, confirming that the methacrylic acid and ethyl acrylate copolymer coating effectively protected the encapsulated drugs during transit through a simulated GI environment (i.e., microfluidic winding path). We observed pH-responsive drug release once the microrobots reached the simulated intestinal environment at the target site. Magnetic guidance in the colon-mimetic microfluidic model enabled the precise localization of the microrobots near the cancer spheroid at the target site. Once released, the PNPs showed enhanced binding affinity to the colon cancer cells. While the dense extracellular matrix of tumor tissues may present physical barriers to the deep interstitial penetration of ∼150-nm nanoparticles (*39*), our multistage system prioritizes maximizing localized retention against luminal washout. This high-density surface localization creates a sustained concentration gradient at the target site, subsequently facilitating both nanoparticle internalization and DOX diffusion into the tumor. Quantitatively, this dual-stage strategy improved nanoparticle retention within cancer spheroid by 3.4-fold and increased cytotoxicity by 4.3-fold compared to nanoparticles administered alone, highlighting the practical advantage of integrating multiscale targeting into oral delivery. Beyond the microfluidic model, the consistent magnetization of individual microrobots also enabled their assembly into chain-like structures through magnetic dipole interactions, providing an effective walking locomotion mode under a rotating magnetic field. This capability was further validated in ex vivo porcine intestine and stomach tissues, where the assembled microrobots showed robust locomotion on compliant and folded GI surfaces. Their ability to traverse tissue folds in both organs shows that the proposed actuation strategy is not limited to planar microfluidic channels and can function in more physiologically relevant GI environments.

Despite these promising results, technical challenges remain. The current manufacturing process, particularly the high-resolution 2PP printing technique, needs optimization for large-scale production to meet practical and clinical requirements. Future research could explore alternative fabrication methods such as roll-to-roll high-resolution 3D printing (*40*) and soft lithography (*41*) to enhance scalability and reduce production costs. In addition, although the PNP strategy enhances cancer-specific interactions, donor-to-donor variations in membrane composition may introduce subtle variability in targeting performance. Addressing this variability through standardized membrane preparation will be essential for future translation to ensure consistent targeting efficiency. Moreover, our colon-mimetic microfluidic model provides a controllable and reproducible environment as a proof of concept rather than a full physiological replica of the in vivo GI environment. Its main strength is that it captures several key barriers relevant to local delivery, including flow-driven washout, path confinement, target-site localization, and release in the vicinity of a tumor-mimetic structure. However, it does not reproduce the full complexity of the GI tract, such as peristaltic deformation, mucus coverage, heterogeneous lumen geometry, or long-distance transit from the site of administration to the disease site. Accordingly, the current experiments primarily validate local magnetic actuation, localized release, and retention under flow, rather than full GI-scale navigation. Future validation in animal models will therefore be essential to assess biodistribution, therapeutic efficacy, and safety.

Overall, this study establishes a proof-of-concept microrobot platform that integrates micro- and nano-technologies through an environment-responsive release mechanism, a fillable chamber–enabled magnetization strategy, and hierarchical targeting across scales. While further development is required before clinical translation, this dual-stage approach offers a foundation for advancing oral delivery strategies to overcome the complex physiological barriers encountered in the human body. This innovative approach holds potential to enhance the precision and efficacy of targeted therapeutic interventions.

## MATERIALS AND METHODS

### Microrobot fabrication, magnetization, cargo loading, and sealing

All 3D models were created using commercial computer-aided design software (Autodesk Inventor 2022), and the model files were exported as STL files. The microrobots were fabricated following the protocol previously reported (*26*). A commercial 2PP printer (Photonic Professional GT2, Nanoscribe, with an immersion objective 10×) was used to fabricate these structures using the commercial resin IP-Q. After printing, the microrobots were cleaned by immersing them in propylene glycol monomethyl ether acetate (PGMEA) for 1 hour and in isopropyl alcohol (IPA) for 10 min. Once thoroughly cleaned, they were dried using nitrogen and postcured under ultraviolet (UV) light (Form Cure, λ = 405 nm) for 2 hours.

A 10% (w/v) polyvinylpyrrolidone (PVP; weight-average molecular weight ∼1,300,000; Sigma-Aldrich, 437190) solution was prepared. An NdFeB microparticle suspension was then prepared by dispersing NdFeB microparticles (mass = 500 mg) in 1 ml of the PVP solution. The magnetic suspension was loaded into the microrobot chambers by centrifugation at 2500 relative centrifugal force (rcf) for 3 min, which drives particle sedimentation into the chamber and promotes formation of a packed particle layer at the chamber bottom. Residual suspension surrounding the microrobots was removed, and the microrobots were dried at 60°C to remove solvent and stabilize the internal magnetic layer. To physically isolate the magnetic layer from the subsequent cargo, a thin PDMS barrier layer was formed as follows: Microrobots were dipped into PDMS resin (10:1) and centrifuged at 2500 rcf for 3 min to drive the PDMS layer to cover the chamber bottom region above the NdFeB layer. The PDMS was cured at 60°C to generate a protective barrier separating the magnetic layer from the drug payload. After formation of the internal NdFeB layer, microrobots were postmagnetized using an impulse magnetizer (M-10-30, ASC Scientific) by applying five magnetization pulses with a peak magnetic field of about 2.5 T to define a consistent magnetization direction across microrobots.

Magnetized microrobots were filled with cargo using a vacuum-assisted loading method. Briefly, a droplet of cargo solution was placed over the microrobot array, and the array was placed in a desiccator connected to a pump. The vacuum pressure facilitated the entry of the solution through the pores on the lid of the microrobots. Once fully loaded, the excess solution was removed by spinning (using a spin coater from Laurell Technologies), and the loaded microrobots were stored in a refrigerator at 4°C to minimize cargo evaporation. The pH-responsive solution was coated onto a glass slide by spinning for 15 s at 400 rpm, then immediately used for dip sealing. A rheometer (Anton Paar, MCR 302) was used to mechanically dip seal the microrobots with the coated pH sealing layer.

### Platelets and membrane derivatives preparation

Human blood containing an acid citrate dextrose solution as an anticoagulant was sourced from Cambridge Bioscience and access to the samples was managed through the Imperial College Healthcare Tissue Bank (ICHTB). ICHTB is approved by Wales REC3 to release human material for research (17/WA/0161). Samples used in this study were issued under subcollection ENG_MS_21_025. The blood was centrifuged at 100*g* for 20 min at room temperature, separating red blood cells (RBCs) and platelet-rich plasma (PRP). The PRP was further centrifuged at 800*g* for 20 min at room temperature to obtain a platelet pellet. This pellet was resuspended in PBS buffer containing 2 μM PGE1 and 1× protease inhibitor (A32955, Thermo Fisher Scientific), then stored at −80°C. All centrifugation steps were performed with minimal acceleration and deceleration to prevent platelet activation during the collection process. Platelet membrane derivatives were produced through three repeated freeze-thaw cycles. After each thaw, the platelet suspensions were centrifuged at 6600*g* for 10 min and washed with PBS buffer containing 1× protease inhibitor. The platelet membrane suspension in water was then tip-sonicated at 20% amplitude with a 2-s on and 1-s off interval for 1.5 min in an ice bath using a tip sonicator (VCX 500, Sonics & Materials Inc.).

### Preparation of PLT membrane– and RBC membrane–coated nanoparticles

DOX hydrochloride (D4193, VWR) was desalted into its free base form. Specifically, 5.5 mg of DOX hydrochloride was dissolved in 1 ml of methanol, and 20 μl of triethylamine (TEA, 471283, Sigma-Aldrich) was added. After stirring overnight at room temperature in the dark and drying under nitrogen (N_2_), the desalted DOX was extracted with 1 ml of chloroform with gentle shaking and collected. This chloroform extraction and N_2_ blowing cycle was repeated to remove any remaining salts, and the dried DOX-free base was stored at −20°C for future use. Using the single emulsion method, DOX-loaded PLGA nanoparticles were synthesized. A mixture of 2 mg of DOX and 4 mg of Resomer RG 502 H, poly(d,l-lactide-co-glycolide) (719897, Merck) was dissolved in 200 μl of dichloromethane (DCM). This solution was emulsified in 1.2 ml of a 5% (w/v) polyvinyl alcohol (PVA, 81381, Sigma-Aldrich) solution in deionized (DI) water using a tip sonicator (VCX 500, Sonics & Materials Inc.) at 30% amplitude with a cycle of 1-s on, 1-s off for 5 min, in an ice bath. This emulsion was then introduced into 4 ml of a 0.5% (w/v) PVA solution in DI water and stirred for 3 hours at room temperature to evaporate the DCM. The emulsion was sonicated again under the same conditions. The resulting water/oil/water emulsion was added dropwise to 8 ml of a 5% (w/v) PVA solution and stirred at room temperature for 3 hours to facilitate further solvent evaporation. To collect the nanoparticles, 35 ml of DI water was added, followed by centrifugation at 20,130*g* for 20 min at 4°C. The nanoparticles were resuspended in DI water with an equal amount of sucrose (A15583, Thermo Fisher Scientific) and freeze-dried for later use. To create PNPs, PLGA nanoparticles and platelet membrane derivatives were mixed in DI water at a membrane–to–core weight ratio of 1:1. This mixture was sonicated at 35% amplitude with a cycle of 2-s on, 1-s off for 2 min and washed via centrifugation at 20,130*g* for 15 min at 4°C. For nanoparticles labeled with 1,1ʹ-dioctadecyl-3,3,3ʹ,3ʹ-tetramethylindodicarbocyanine, 4-chlorobenzenesulfonate salt (DiD, excitation 644 nm/emission 665 nm, V22887, Thermo Fisher Scientific) and containing DOX, 0.1 wt % of DiD relative to PLGA weight was added to the DOX in DCM. Rhodamine 6G–loaded nanoparticles were prepared by substituting an equivalent weight of Rhodamine 6G (Sigma-Aldrich, 252433) for DOX in DCM, following the same nanoparticle preparation procedures as described above. RBC membrane–coated nanoparticles were prepared using the same method as PNPs.

### PNP characterization

The size and surface zeta potential of nanoparticles and PNP-Vs were assessed via DLS using a Malvern Zetasizer Nano-ZS. For TEM analysis, samples were adsorbed and dried on carbon-coated 400-mesh copper grids (CF400-CU-50, Electron Microscopy Sciences) at room temperature, negatively stained with 1 wt % uranyl acetate (22400, Electron Microscopy Sciences) for 30 s, and imaged with a TEM (JEM-2100Plus, JEOL Ltd). To confirm platelet membrane coating integrity on PNPs post–cell internalization, 3,3′-dioctadecyloxacarbocyanine perchlorate (DiO)–stained PLT-Vs were first prepared. DiO dye (1 μM) was mixed with a PLT-V suspension in PBS for 1 hour at 37°C. Excess DiO dye was washed away, and DiO-stained PLT-Vs were collected using an Amicon 10-kDa molecular weight cutoff tube (UFC901008, Merck). DiD–labeled PLGA nanoparticles were coated with DiO-stained PLT-Vs as previously described. Prepared nanoparticles (200 μg/ml) were incubated with HCT116 cells at 37°C for 4 hours, washed with PBS, and fixed with 4% (w/v) paraformaldehyde in PBS, and the nuclei were stained with Hoechst 33342. Fluorescent images were captured using a confocal microscope (SP8, Leica). For the determination of drug loading yield, weighted PNPs were dissolved in 1 ml of dimethyl sulfoxide (276855, Sigma-Aldrich) using bath sonication for 30 min. After centrifugation at 20,133*g* for 15 min, the DOX in the supernatant was collected, dried, and resuspended in 10 ml of Dulbecco’s phosphate-buffered saline (DPBS).

### Western blotting of platelet membrane proteins

Western blot analyses were used to confirm the inheritance of platelet membrane proteins to BNPs. Before protein extraction, PNPs (approximately 500 μg of nanoparticle mass) were washed three times with PBS buffer by centrifugation at 20,130*g* for 15 min at 4°C to remove unbound proteins. Protein samples from lysed platelets, PLT-Vs, and PNPs were prepared with a minimum concentration of 1.5 mg/ml using radioimmunoprecipitation assay buffer (ab156034, Abcam) with 1× protease inhibitor. Protein concentrations were measured with a bicinchoninic acid assay kit (23227, Thermo Fisher Scientific). Protein samples (20 μg) mixed with sample loading buffer (1610747, Bio-Rad) containing 5% 2-mercaptoethanol (1610710, Bio-Rad) were denatured at 95°C for 5 min and loaded per well. As 8 μl of prestained protein standard (1610374, Bio-Rad) was loaded for molecular weight reference in each gel, proteins were separated on 4 to 20% Mini-PROTEAN TGX Stain-Free Precast Gels (4568096, Bio-Rad) in a running buffer (1610732, Bio-Rad). Electrophoresis was performed at 80 V for the stacking gel and 100 V for the resolving gel. The separated proteins were transferred to polyvinylidene fluoride membranes (1704157, Bio-Rad) using the Trans-Blot Turbo Transfer System (1704150, Bio-Rad) according to the manufacturer’s instructions. After membrane blocking with 1% bovine serum albumin (A2153, Sigma-Aldrich) in tris-buffered saline (TBS, 1706435, Bio-Rad) with 0.01% Tween 20 (1706531, Bio-Rad) (TBST), membranes were incubated with primary antibodies specific for Integrin α6 (3750, Cell Signaling Technology), Integrin β3 (13166S, Cell Signaling Technology), and P-selectin (84298S, Cell Signaling Technology) at a 1:1000 dilution overnight at 4°C. Then, IRDye 680RD Goat anti-Rabbit IgG Secondary Antibodies (926-68071, LI-COR) at 1:15,000 were incubated at room temperature for 1 hour, and images were acquired using the Odyssey CLx Imager (LI-COR).

### In vitro microfluidic colon cancer model and ex vivo locomotion evaluation

The microfluidic chip consists of a villi-like substrate and channel, both made of PDMS (10:1) using a molding technique. The villi-like substrate mold was created using a 2PP printer, while the channel mold was fabricated with a 3D printer (Prusa SL1S).

Before cell seeding, the fully assembled microfluidic chip was sterilized using UV irradiation for 1 hour. The channel was coated with fibronectin (50 μg/ml, F1141, Merck) for 1 hour. After coating, the channel was washed with DPBS, and HCOEPCs (2 × 10^6^ cells/ml) were introduced into the channel, allowing them to adhere for 1 hour at 37°C in a 5% CO_2_ atmosphere. Unattached cells were removed by washing with cell culture medium, and the HCOEPCs were cultured for an additional 48 hours to form a stable epithelial layer. For spheroid formation, 2 × 10^4^ HCT116 cells were seeded into a U-bottom 96-well plate (174925, Thermo Fisher Scientific) and cultured for 5 days until spheroids formed. One spheroid was then transferred to the target site on the microfluidic platform and incubated for an additional 24 hours to facilitate attachment to the epithelium. The coculture medium was a 1:1 mixture of normal epithelial cell culture medium and cancer cell culture medium (chip medium). The chip medium was perfused through the microfluidic chip using hydrostatic pressure–driven flow every 24 hours. All incubations were performed at 37°C in a 5% CO_2_ atmosphere.

Microrobot locomotion was actuated using an external magnetic field generator (MFG-100-I, Magnebotix). For rotating-field actuation, a uniform rotating magnetic field with magnitude 5 mT was applied at a rotation frequency of 1 Hz. Ex vivo locomotion was evaluated using porcine stomach and intestinal tissues to assess the mobility of the proposed microrobot system on physiologically relevant, compliant, and folded GI surfaces.

### Adhesion assay to normal colon epithelial cells and cancer cells

HCOEPCs, SW480, and HCT116 cells (2 × 10^6^) were seeded in separate six-well plates and cultured overnight. The cells were then exposed to a solution with DiD-labeled lyophilized nanoformulations (200 μg/ml) at 4°C for 30 min in their respective culture media. After three washes with PBS to remove unbound nanoparticles, cells were incubated at 37°C for 2 hours to allow for the bound nanoparticles to be internalized. The cells were detached by treating with 0.5 mM ethylenediaminetetraacetic acid (EDTA, 15575020, Thermo Fisher Scientific) and then collected after centrifugation at 200*g* for 5 min. They were subsequently fixed with 4% paraformaldehyde for 10 min. The pellets were washed once with flow cytometry solution [PBS with 2% (v/v) FBS] and then resuspended in 500 μl of the flow cytometry solution. The fluorescence intensity of the nanoparticles within the cells was measured using a BD LSRFortessa Cell Analyzer, with the mean fluorescence intensity data analyzed using FlowJo version 10 software. PNPs were preblocked with anti–P-selectin antibody to validate platelet membrane–mediated targeting. Briefly, PNPs (1 mg/ml) were preincubated with anti–P-selectin antibody (20 μg/ml; 14-0628-82, Thermo Fisher Scientific) for 1 hour at room temperature and then washed with PBS to remove excess antibody.

### Cytotoxicity measurement using CCK-8

Cytotoxicity was assessed using the CCK-8 (Sigma-Aldrich, 96992). HCOEPC, SW480, and HCT116 were seeded at a density of 10,000 cells per well. After 24 hours of treatment with various concentrations of DOX-loaded nanoparticles, the medium was replaced with 100 μl of fresh medium. Subsequently, 10 μl of CCK-8 solution was added, and the cells were incubated for 3 hours at 37°C with 5% CO_2_. Absorbances were measured at 450 and 650 nm using a plate reader (SpectraMax M5, Molecular Devices), and cell viability was calculated from the data (*n* = 3 independent experiments and *n* = 3 technical replicates). For washout assays designed to evaluate targeting-dependent cytotoxicity, cells were first exposed to PNP-DOXs for 30 min to allow nanoparticle binding. The medium was then replaced to remove unbound nanoparticles, followed by incubation for an additional 24 hours before performing the CCK-8 assay as described above.

### In vitro demonstration of targeting and anticancer efficacy

DiD-labeled BNP-DOXs and PNP-DOXs were prepared at a 4.2 μg/ml concentration in chip medium. Microrobots containing 0.42 μg of PNP-DOX were also prepared. A volume of 200 μl of DiD-labeled nanoparticles or microrobots was introduced into the colon epithelium–coated microchannel under hydrostatic pressure–driven flow for 30 min, with pressure resets every 10 min. Unbound nanoparticles or microrobots were washed away with DPBS for 30 min, followed by an additional washing step 12 hours later. The treatment continued for a total of 24 hours. DiD expression was imaged using a microscope (AMF4300, EVOS) at the colon cancer spots. Intensity was measured using ImageJ software. To quantify the delivered payload on a per-spheroid basis, individual cancer spheroids were collected and dissociated by collagenase IV (1 mg/ml; 17104-019, Thermo Fisher Scientific) at 37°C for 15 min. Cells were pelleted by centrifugation at 300*g* for 5 min and lysed with 100 μl of lysis buffer [50 mM tris-HCl, 150 mM NaCl, 0.1% Triton X-100, and DNase I (10 U/ml)]. Lysates were centrifuged at 1000*g* for 5 min at 4°C to remove cellular debris, and the supernatant was further centrifuged at 20,000*g* for 30 min at 4°C to concentrate nanoparticles. The nanoparticle-enriched pellet was resuspended in 200 μl of PBS, and DOX absorbance was quantified using a plate reader (SpectraMax M5, Molecular Devices) at a 485-nm wavelength. The retained nanoparticle payload per spheroid was calculated using a calibration curve generated from DOX-loaded nanoparticles with known concentrations and subsequently normalized to the administered dose. To analyze anticancer efficacy, the microfluidic platforms were stained with 1 μM Calcein AM and 4 μM ethidium homodimer–1 for anticancer efficacy for 15 min at 37°C in a 5% CO_2_ atmosphere and washed; then, green and red fluorescence were imaged using a microscope (AMF4300, EVOS), and the ratio of red to green fluorescence intensity was calculated using ImageJ software to assess the cytotoxicity.

### Statistical analysis

The number of independent replicates is specified in the figure legend for each figure. Statistical comparisons between two groups were conducted using a two-tailed unpaired Student’s *t* test. One-way analysis of variance (ANOVA) was used for comparisons among three or more groups, followed by Tukey’s post hoc tests. Data were analyzed using Prism 8.0.1 software (GraphPad) and are presented as mean ± SD. Statistical significance is indicated by *, **, or ***, corresponding to *P* values of <0.05, <0.01, and <0.001, respectively; *P* values > 0.05 are considered not significant (N.S.).
