# Supplementary material for: Multistage microrobots with pH-responsive release of platelet membrane–coated nanoparticles
Source: Sci Adv. 2026 Jul 15;12(29):eaee6534. doi: 10.1126/sciadv.aee6534 (PMC13371902; doi:10.1126/sciadv.aee6534)
Supplement: Supplementary file 1 — Figs. S1 to S10 Supplementary Methods Legends for movies S1 to S3 [file sciadv.aee6534_sm.pdf]

Science Advances
AAAS

Supplementary Materials for

Multistage microrobots with pH-responsive release of platelet membrane–coated nanoparticles

Rujie Sun *et al.*

Corresponding author: Molly M. Stevens, molly.stevens@dpag.ox.ac.uk

Sci. Adv. **12**, eaee6534 (2026)
DOI: 10.1126/sciadv.aee6534

The PDF file includes:

Figs. S1 to S10
Supplementary Methods
Legends for movies S1 to S3

Other Supplementary Material for this manuscript includes the following:

Movies S1 to S3

Supplementary Figures

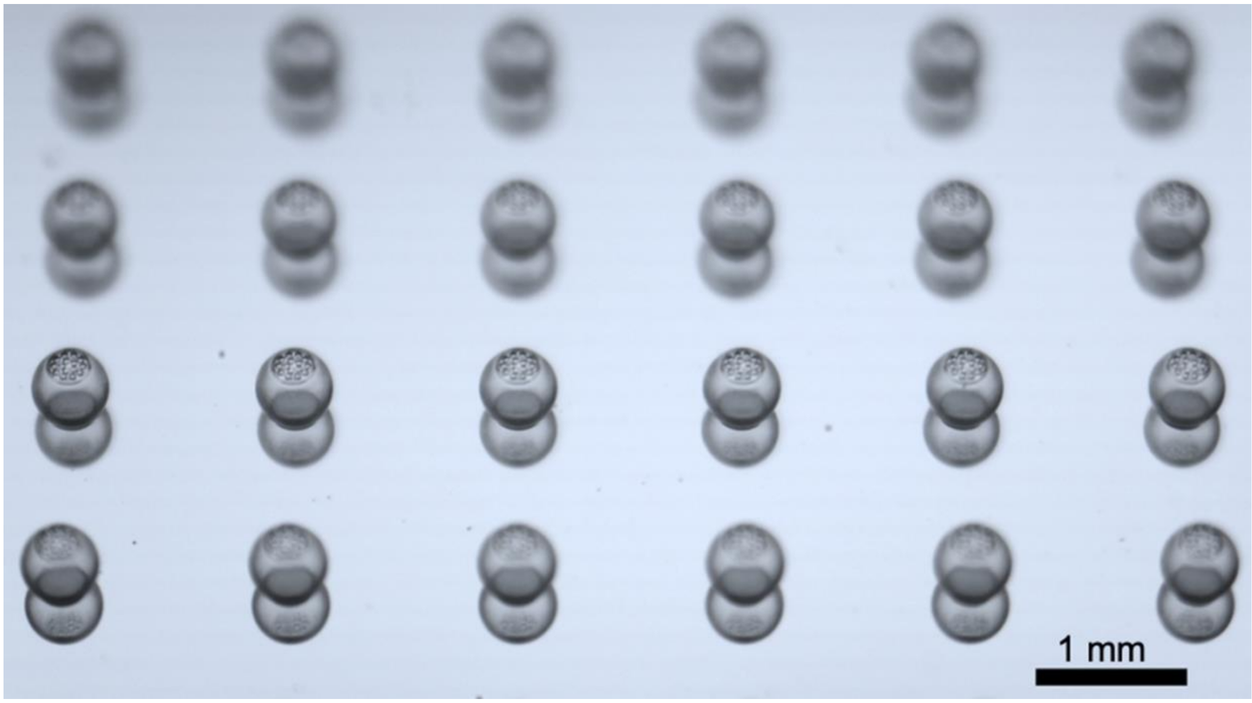

Fig. S1. Optical image of the printed microrobot array, showcasing the uniformity and structural details of the fabricated microrobots

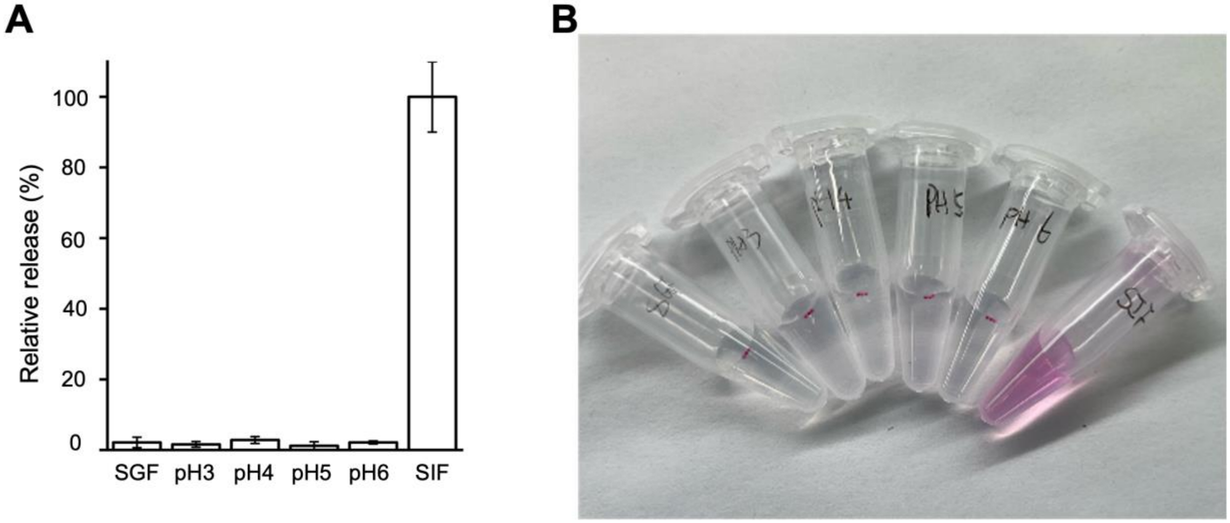

Fig. S2. Release characterization of RhB-loaded, dip-sealed microrobots after 1 hour incubation in SGF, SIF, and solutions at pH 3, 4, 5, and 6. (A) Relative release quantified from the absorbance of the surrounding solution. (B) Photographs of the corresponding incubation solutions, showing negligible release in SGF and pH 3–6, but substantial release in SIF. From left to right: SGF, pH 3, pH 4, pH 5, pH 6, SIF. (n = 3 independent samples).

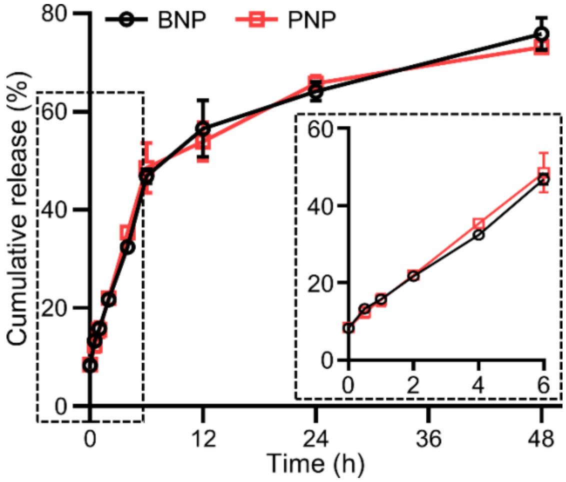

Fig. S3. Doxorubicin release profile from BNP-DOXs and PNP-DOXs in PBS at 37 °C over time (n = 3 independent samples).

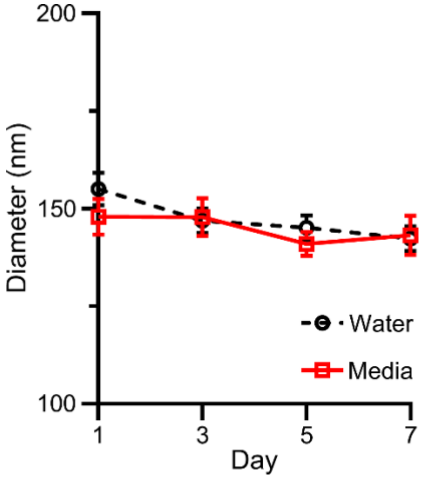

Fig. S4. Colloidal stability of PNPs in D.I. water and cell culture medium over 7 days at 4 °C (n = 3 independent samples, n =3 technical replicates).

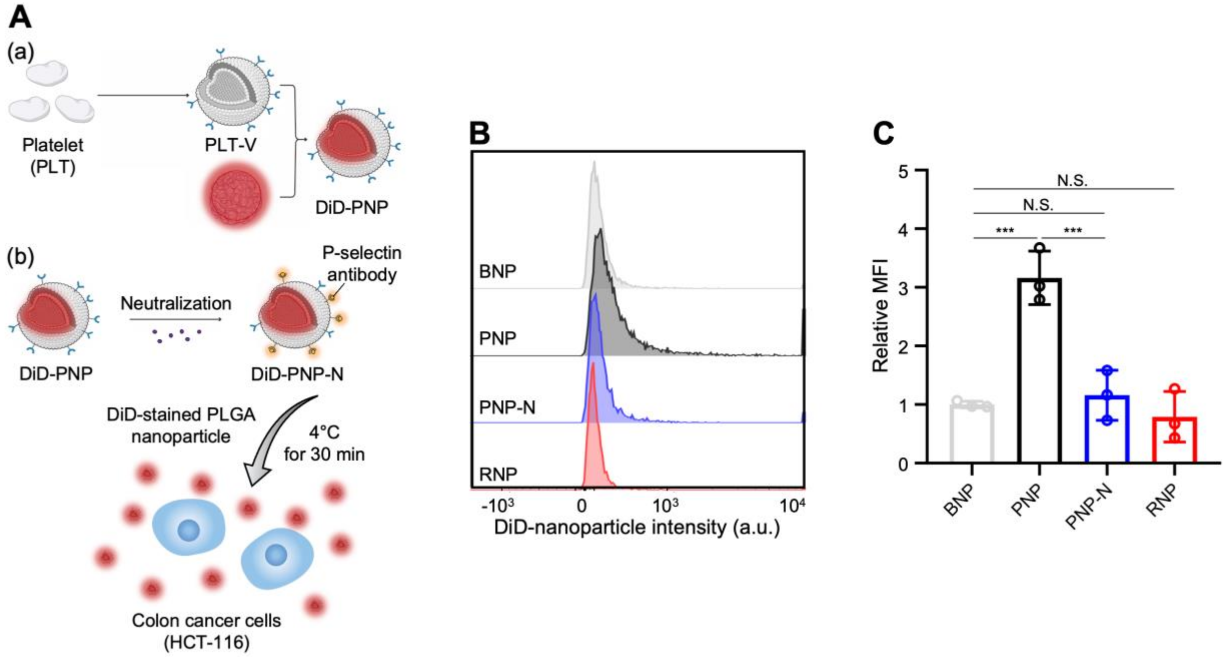

Fig. S5. Receptor-blocking assay validating platelet membrane-mediated tumor targeting. (A) Schematic illustration of the experimental design for evaluating platelet membrane-coated nanoparticle targeting. DiD-labeled platelet membrane-coated nanoparticles (PNPs) were prepared from platelet membrane vesicles (PLT-V) and PLGA nanoparticles. To assess the role of P-selectin–mediated interactions, PNPs were pre-treated with an anti-P-selectin antibody to generate neutralized nanoparticles (PNP-N). DiD-labeled PLGA nanoparticles (BNP) and red blood cell membrane-coated nanoparticles (RNP) were used as controls. Nanoparticle binding to HCT116 colon cancer cells was assessed after incubation at 4 °C for 30 minutes. (B) Representative flow cytometry histograms showing DiD fluorescence intensity of HCT116 cells after exposure to each nanoparticle formulation. (C) Flow cytometry-based 2D in vitro nanoparticle adhesion assay on HCT116 cells, showing relative mean fluorescence intensity (MFI) normalized to the BNP condition in each group (n = 3 independent samples). Data are presented as mean ± standard deviation (S.D.). One-way ANOVA with posthoc Tukey's test was used to compare differences among different conditions (C); N.S., non-significant; ***, $p < 0.001$. Figure A was created in BioRender. Cho, Y. (2026) https://BioRender.com/n3qfhgp.

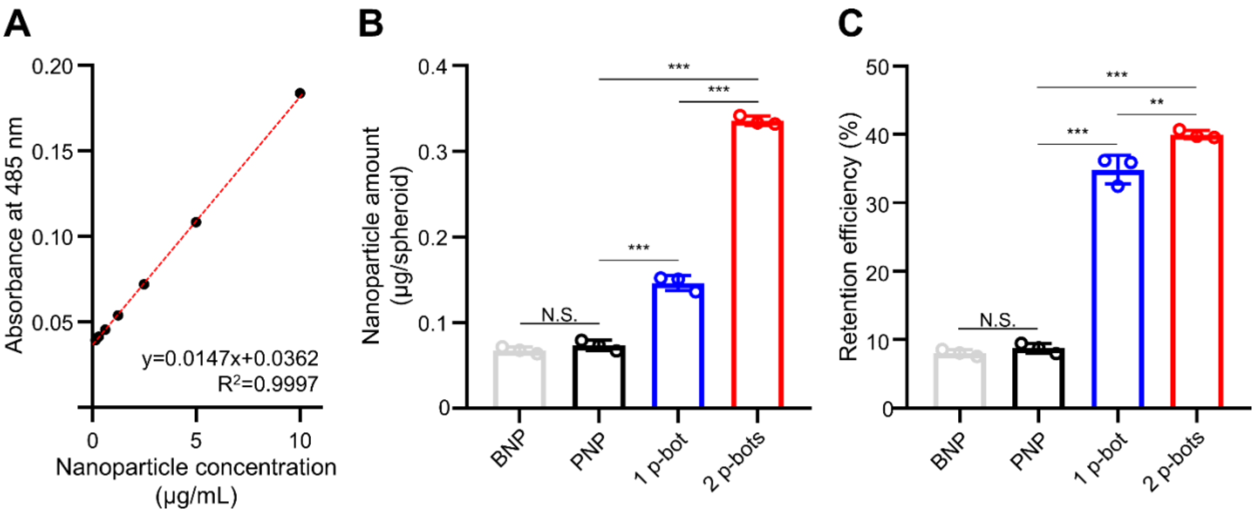

Fig. S6. Quantitative estimation of doxorubicin-loaded nanoparticle payload delivered to individual cancer spheroids. (A) Calibration curve correlating nanoparticle concentration with absorbance at 485 nm. The linear relationship was used to estimate the amount of nanoparticles recovered from individual spheroids. (B) Quantification of nanoparticle amount recovered from individual spheroids after treatment with BNP, PNP, 1 p-bot, or 2 p-bots (BNP, PNP, and 2 p-bots: 0.84 μg administered dose and 1 p-bot: 0.42 μg administered dose). (C) Retention efficiency calculated as the percentage of the administered nanoparticle dose recovered from each spheroid. ($n = 3$ independent chips) Data are presented as mean ± standard deviation (S.D.). One-way ANOVA with post-hoc Tukey's test was used to compare the difference among different conditions (B, C); N.S., non-significant; **, $p < 0.01$; ***, $p < 0.001$.

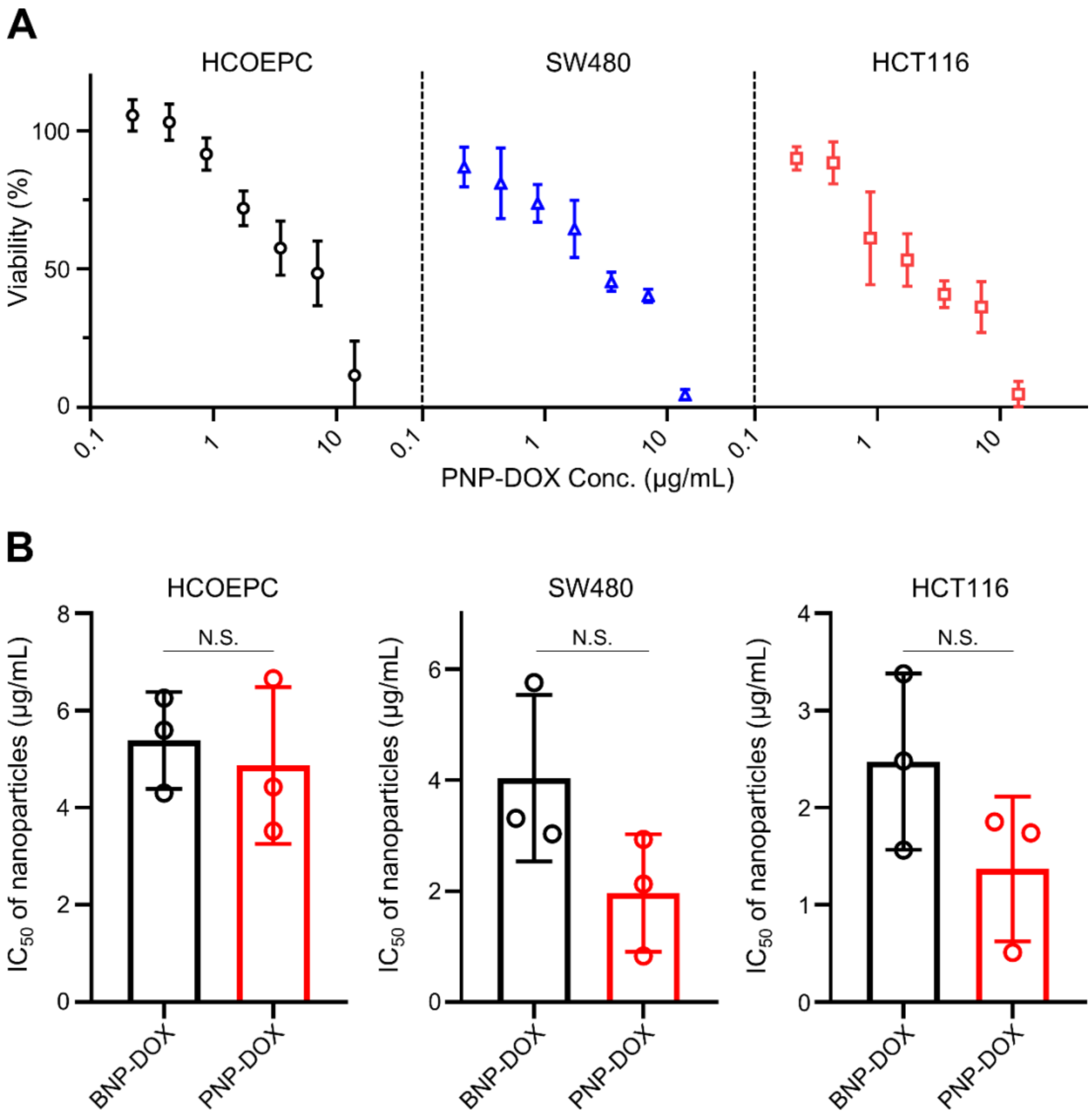

Fig. S7. Cytotoxicity of doxorubicin-loaded nanoparticles under static incubation conditions. **(A)** CCK-8 analysis of normal colon epithelial cells (HCOEPC) and colon cancer cell lines (SW480 and HCT116) treated with PNP-DOX at various concentrations for 24 hours (n = 3 independent samples). (B) IC_{50} values of BNP-DOX and PNP-DOX in HCOEPC, SW480, and HCT116 cells after 24-hour treatment. (n = 3 independent measurements). Data are presented as mean ± standard deviation (S.D.). The two-tailed unpaired Student's t-test was used to compare the means of two groups; N.S., non-significant.

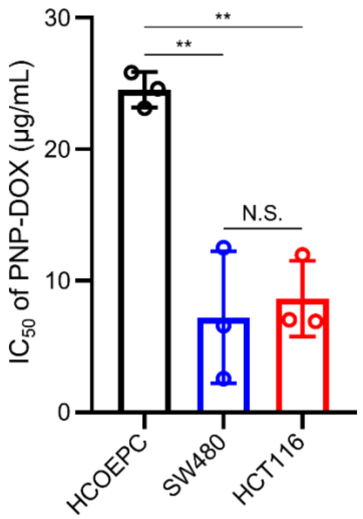

Fig. S8. IC_{50} values of normal colon epithelial cells (HCOEPC) and colon cancer cell lines (SW480 and HCT116) under different concentrations of PNP-DOX after 30-minute targeting, followed by washout and 24-hour incubation in CCK-8 assays. (n = 3 independent samples). Data are presented as mean ± standard deviation (S.D.). One-way ANOVA with posthoc Tukey's test was used to compare differences among different conditions; N.S., non-significant; **, $p < 0.01$.

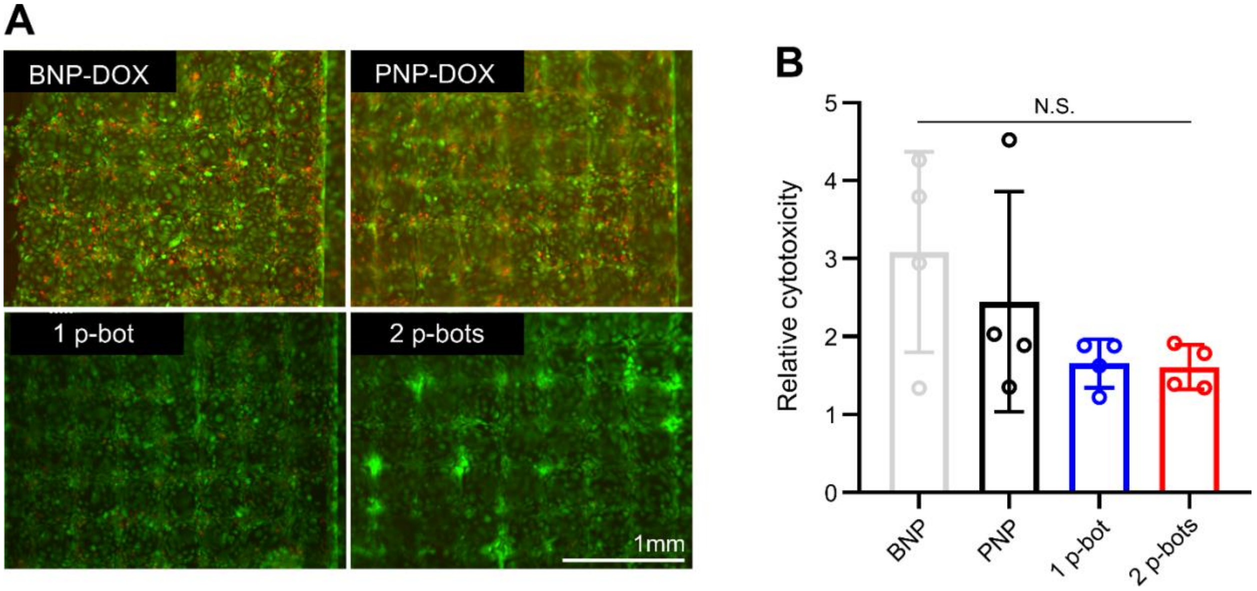

Fig. S9. Off-target side effect assay of p-bot multiscale delivery using colon-mimetic microfluidic platforms. (A) Fluorescent microscopy images of viability staining on normal colon epithelium in the colon-mimetic microfluidic platform (Green: Calcein AM, Red: Ethidium homodimer-1). (B) Relative cytotoxicity comparison of normal colon epithelium in the colon-mimetic microfluidic platform after 24 hours of various drug administrations, normalized to the non-treated conditions (n = 4 independent chips). Data are presented as mean ± standard deviation (S.D.). One-way ANOVA with post-hoc Tukey's test was used to compare the difference among different conditions (B); N.S., non-significant.

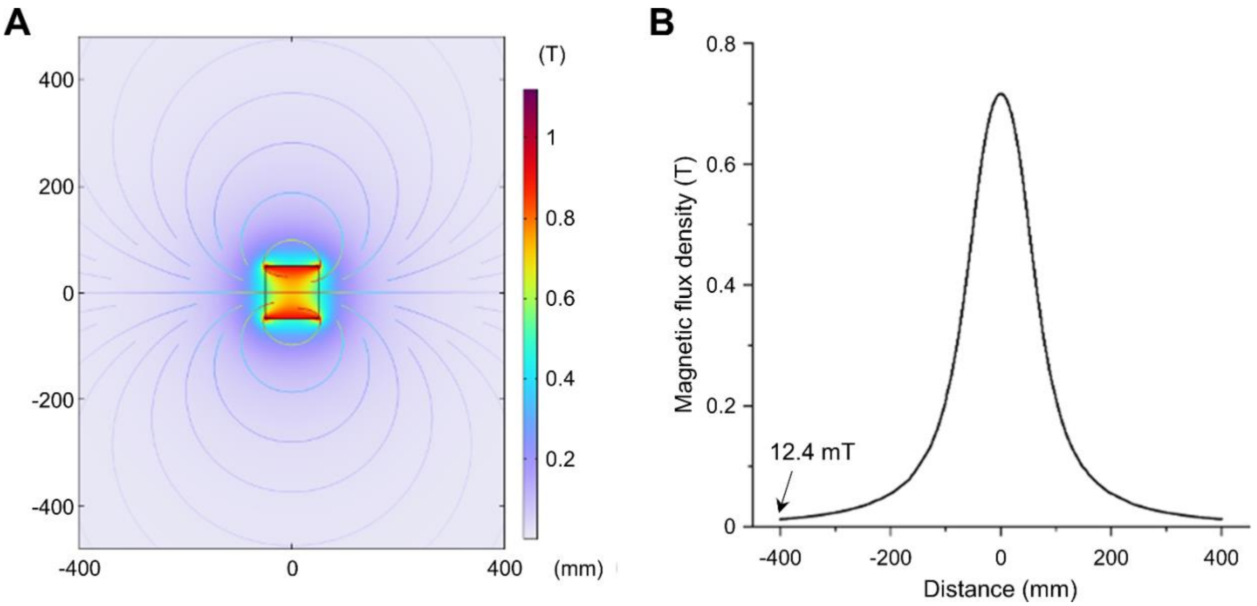

Fig. S10. Simulation of magnetic field generation using a permanent magnet. (A) Simulated spatial distribution of magnetic flux density around the permanent magnet. (B) Magnetic flux density as a function of distance along the central axis, showing a gradual decay with distance while remaining at 12.4 mT over 400 mm. Distance is measured from the magnet center along the central axis.

Supplementary methods

Materials

All chemical reagents were used without further purification. IP-Q (Nanoscribe) was used as the printing resin. Acetone (VWR 20066.330) and pure water (Millipore Milli-Q Integral 3) were used to clean the substrate. Propylene glycol monomethyl ether acetate (PGMEA, Sigma-Aldrich 484431) and isopropanol (IPA, Fisher Chemical P/7500/17) were used to remove the non-polymerized resin. A silicon wafer (Nanoscribe) was used as the printing substrate. Rhodamine B (Sigma-Aldrich R6626) and Rhodamine 6G (Sigma-Aldrich, 252433) were used as a loading cargo for the drug release demonstration. Pluronic® F-127 (Sigma-Aldrich P2443-250G) was used as a copolymer for the cargo solution. Phosphate-buffered solution (PBS, Gibco,1X) was used for release studies. Kollicoat® MAE 30 DP (Sigma-Aldrich 42385-500G) was used for sealing. DOW SYLGARD® 184 (Farnell 101697) was used to build the microfluidic chip.

Platelets (PLTs) and membrane derivatives preparation

Human blood containing an acid citrate dextrose (ACD) solution as an anticoagulant was sourced from Cambridge Bioscience and access to the samples was managed through the Imperial College Healthcare Tissue Bank (ICHTB). ICHTB is approved by Wales REC3 to release human material for research (17/WA/0161). Samples used in this study were issued under sub-collection ENG_MS_21_025. The blood was centrifuged at 100g for 20 minutes at room temperature, separating red blood cells (RBCs) and platelet-rich plasma (PRP). The PRP was further centrifuged at 800g for 20 minutes at room temperature to obtain a platelet pellet. This pellet was resuspended in PBS buffer containing 2 μM PGE1 and 1x protease inhibitor (A32955, ThermoFisher Scientific), then stored at -80 °C. All centrifugation steps were performed with minimal acceleration and deceleration to prevent platelet activation during the collection process. Platelet membrane derivatives were produced through three repeated freeze-thaw cycles. After each thaw, the platelet suspensions were centrifuged at 6,600g for 10 minutes and washed with PBS buffer containing 1x protease inhibitor. The platelet membrane suspension in water was then tip-sonicated at 20% amplitude with a 2-second on and 1-second off interval for 1.5 minutes in an ice bath using a tip sonicator (VCX 500, Sonics & Materials Inc.).

Human red blood cell (RBC) isolation and RBC membrane derivative preparation

RBCs were isolated from whole blood by centrifugation at 100 g for 20 minutes at room temperature. The collected RBCs were washed with cold phosphate-buffered saline (PBS), and subsequently lysed using a hypotonic solution (0.25x PBS with deionized water) in an ice bath for 30 minutes, with vortexing every 10 minutes to facilitate hemolysis. The hemolyzed RBCs were collected by centrifugation at 17,000 g for 10 minutes at 4 °C. The resulting membrane fraction was further processed through repeated freeze-thaw cycles, followed by tip-sonication for 90 seconds (20% amplitude, 2 seconds on, 1 second off) to obtain RBC membrane vesicles.

Drug loading, release, and stability characterization of nanoparticles

The loading capacity was calculated as (Doxorubicin content from nanoparticle) / (Mass of nanoparticle) × 100. The drug release profile was analyzed by dialyzing 500 μL of BNPs and PNPs (2 mg/mL) in 9.5 mL of PBS using Slide-A-Lyzer MINI Dialysis devices with a 3.5 kDa MWCO

(88400, ThermoFisher Scientific) on a shaker at 37 °C. 500 µL of PBS was collected at each time point and released doxorubicin was collected after centrifugation at 20,133g for 15 minutes. Doxorubicin concentrations were measured using a plate reader (SpectraMax M5, Molecular Devices) with an excitation wavelength of 505 nm and an emission wavelength of 565 nm to determine the drug loading capacity and release profile (n = 3 independent measurements). Long-term stability in water and media was assessed by measuring size changes of 1 mg/mL PNPs in water and DMEM (11965092, Gibco) with 10% (v/v) fetal bovine serum (FBS, 10082147, ThermoFisher Scientific) and 1% penicillin-streptomycin (15140122, Gibco) over 7 days at 4 °C.

Cell culture

All cells were cultured at 37 °C in a 5% CO_2 atmosphere. Normal human colon epithelial cells (HCOEPCs, 10HU-096, iXCells Biotechnologies) were maintained in epithelial cell growth medium (MD-0041, iXCells Biotechnologies). Human colon cancer cell lines SW480 (CCL-228, ATCC) and HCT116 (CCL-247, ATCC) were cultured in Dulbecco's Modified Eagle Medium (DMEM) (11965092, Gibco) supplemented with 10% (v/v) FBS (10082147, ThermoFisher Scientific) and 1% penicillin-streptomycin (15140122, Gibco).

Magnetic field simulation

Magnetostatic simulations were performed in COMSOL Multiphysics 6.3 using the AC/DC Module. A simplified 2D model was used, consisting of a single NdFeB N52 permanent magnet with dimensions of 100×100 mm, placed in a surrounding air domain. A 2D field map was used to visualize the magnetic flux density, and a line profile along the central axis was extracted to determine the variation of magnetic flux density with distance.

Supplementary videos

Movie S1.
In vitro evaluation of microrobot locomotion in a colon-mimetic microfluidic platform
Movie S2.
Ex vivo evaluation of microrobot locomotion in a porcine stomach
Movie S3.
Ex vivo evaluation of microrobot locomotion in a porcine intestine
